# Supplementary material for: Large impact cratering during lunar magma ocean solidification
Source: Nat Commun. 2021 Sep 14;12:5433. doi: 10.1038/s41467-021-25818-7 (PMC8440705; doi:10.1038/s41467-021-25818-7)
Supplement: Supplementary file 1 — Supplementary Information [file 41467_2021_25818_MOESM1_ESM.pdf]

Supplementary Notes:

Large impact cratering during lunar magma ocean solidification

K. Miljković[1*], M.A. Wieczorek[2], M. Laneuville[3], A. Nemchin[1], P.A. Bland[1], and M.T. Zuber[4]

[1]Curtin University, School of Earth and Planetary Science, Space Science and Technology Centre, Perth, Australia; [2]Université Côte d'Azur, Observatoire de la Côte d'Azur, CNRS, Laboratoire Lagrange, France; [3]Earth-Life Science Institute, Tokyo, Japan; [4]Massachusetts Institute of Technology, Cambridge, USA. *email: katarina.miljkovic@curtin.edu.au

Table of Contents:

Supplementary Figures 1-10

Supplementary Table 1: GRAIL-derived properties of the impact basins

Supplementary Table 2: Summary of chosen numerical input parameters for the target layers

Supplementary Note 1 Numerical impact modelling: Setup

Supplementary Note 2 Numerical impact modelling: Results

Supplementary Note 2.1 Topographic features in basins

Supplementary Note 2.2 Basin morphology with respect to basin size

Supplementary Note 2.3 Basin morphology with respect to the melt layer thickness

Supplementary Note 2.4 Basin morphology with melt layer with different crustal thicknesses

Supplementary Note 2.5 Basin crustal thickness signature as a function of basin size and melt layer thickness

Supplementary Note 2.6 Best-fit scenario for the formation of the South-Pole Aitken basin

Supplementary References

Supplementary Table 1. GRAIL-derived properties of the impact basins shown in Fig. 1.

Impact basin name	Ambient pre-impact crustal thickness[1] (km)	Main rim radius (km)	Inner ring radius (km)
Nectaris	29	442	220
Orientale	36	468	240
Fecunditatis	29	[345]	{172}
Nubium	33	[345]	
Australe	34	{440}	

[1]Using Model 1 of ref. (2).
[] denotes a possible ring[1].
{} denotes a ring location suggested from scaling relations[1].

Supplementary Note 1: Numerical impact modelling setup

The numerical impact simulations assumed a simplified three-layered target that was assumed flat for projectiles smaller than 90 km in diameter and curved for projectiles larger or equal to 90 km in diameter. The layers consisted of (a) a crust that was 10, 25 or 50 km thick, (b) an extremely low viscosity layer with a thickness of 10, 25 or 50 km (from here on referred to as the melt layer), and (c) a solid mantle. The justification of the crustal thickness range comes from the GRAIL-derived crustal thickness map of the Moon[2] (Supplementary Table 1) and estimates of the thickness of the flotation crust from LMO solidification calculations[3,4]. The presence of even the smallest melt fraction in rocks is known to significantly decrease their viscosity[5,6]. We do not address the chemistry of the melt, which determines its solidus temperature, because the melt composition is uncertain and changes as the magma ocean solidifies.

Two temperature profiles were employed for the majority of our simulations. For the first (Initial Moon A in Supplementary Fig. 1), the temperature gradient at the surface was set to 50 K/km, and the temperature was limited to maximum value of 1400 K at 25 km depth to ensure that the mantle was everywhere below the solidus temperature. Below 25 km depth, the mantle temperature profile followed an adiabat. Though this model has a surface temperature of about 80 K (which is somewhat lower than the true average value), however, the near-surface temperature rises to 280 K within 4 km depth due to the steep gradient applied. We note that iSALE does not provide much freedom for customizing temperature profiles when working with curved surfaces. This temperature profile was used for simulating the largest basins, for which size the curved surface plays a role in basin formation[7,8]. Regardless, tests using a Cartesian geometry showed that this lower surface temperature had no significant impact on the final basin morphology when compared with the other tested temperature profiles (Supplementary Fig. 3a). For our second temperature profile, we used the profile H-0LB of Laneuville et al.[9] at 4.5 Ga which is the initial condition for their post-magma ocean thermal evolution simulations. This temperature profile (Initial Moon B in Supplementary Fig. 1) is cooler in the crust and hotter in the mantle than the first temperature

profile. This thermal profile was used for small to mid-size basins, where the target was assumed flat.

Although the two initial temperature profiles described above were used for the majority of our simulations, to further test how the choice of the temperature profile affects the final basin morphology, we also tested a much hotter temperature profile (Initial Moon C in Supplementary Fig. 1). This temperature profile is representative of the interior of the Procellarum KREEP Terrane at 4 Ga[9,10]. Though the temperature of the crust is between our two previous profiles, the uppermost mantle is considerably hotter and exceeds the mantle solidus. Tests show that even when the hottest thermal profile was used, it was the presence or absence of a low viscosity melt layer that played the most dominant role in the final crater morphology. Supplementary Fig. 3 shows that our final crustal thickness profiles are nearly the same for all three temperature profiles. We further investigated how much impact melt was generated using each of these temperature profiles, and found that there was little difference in the size and extent of the melt pool (see, Supplementary Fig. 4).

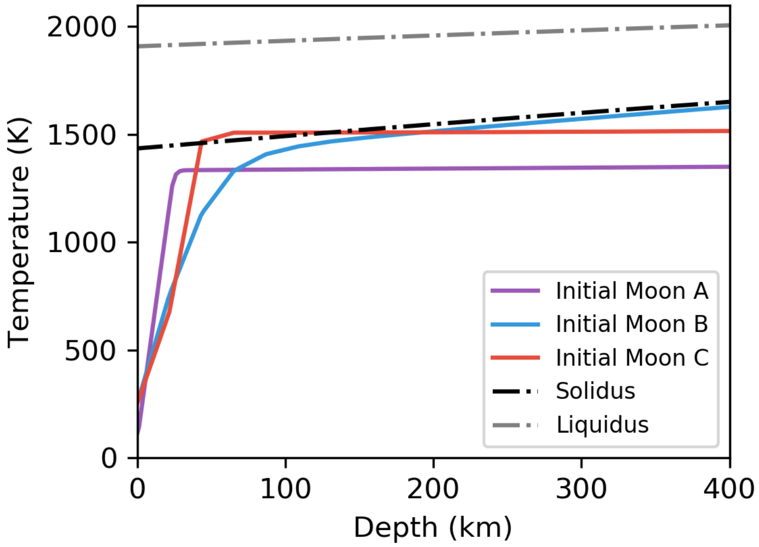

Supplementary Fig. 1: Temperature as a function of depth below the surface. Initial Moon A and B are two thermal profiles used in this work that are representative of the period immediately following magma ocean crystallization. Neither profile has temperatures above the material's solidus. Initial Moon C is the hottest tested temperature profile, which is representative of the interior of the nearside Procellarum KREEP Terrane at 4 Ga. Solidus and liquidus appropriate for dunite[9] are shown in black and gray, respectively.

Supplementary Fig. 2 shows our computed yield strength as a function of depth for the three temperature profiles A, B and C. The panel on the left shows the yield strength profile with depth when there is no melt layer, for the two thermal profiles used in this work (Initial Moon A and B). Considering that these thermal profiles do not cross the set solidus, the yield strength is non-zero. This is changed when we introduce a melt layer, which causes the yield strength to drop to zero where the melt layer is located (Supplementary Fig. 2 right). The right panel considers the presence

of a 25 km thick melt layer, under 25 and 50 km crustal thickness (blue lines) for the thermal case B, and for the thermal profile A with 25 km thick crust (purple line). The thermal profile C, however, crosses solidus and that causes natural formation of a melt layer at depth. The crustal thickness was 25 km but the melt was initiated slightly deeper for approximately 30 km.

The melt layer was treated as a cohesionless fluid with constant low viscosity. Because of the melt being treated as a cohesionless fluid, its yield strength is zero in the computations. Solomatov (2007)[10] suggests that a typical viscosity of near-liquidus materials in a magma ocean is 0.01 Pa s with a factor of 10 uncertainty. Our tests showed that the chosen viscosity of the melt layer had little influence on the final basin morphology for all values less than about 10^{10} Pa s, which is significantly lower than the viscosity of a solid rock. In this work, we used a constant viscosity of 100 Pa s, simply to avoid using (near) zero values in calculation.

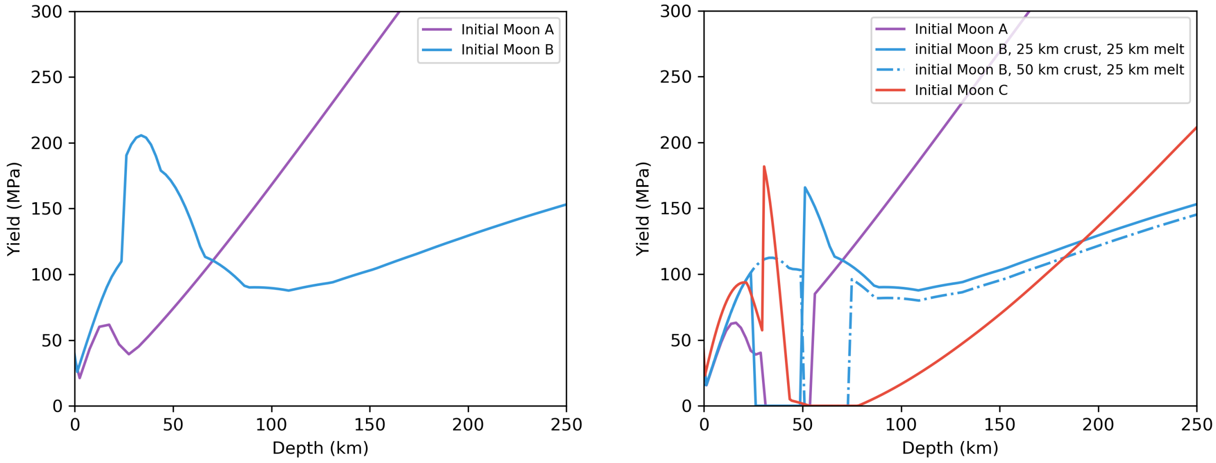

Supplementary Fig. 2: Yield strength profiles as a function of depth below the surface. (left) Yield strength predicted using the two temperature profiles that do not include melt in the mantle (Initial Moon A and B). (right) Yield strength profiles after introducing a 25 km thick melt layer beneath the crust for the three different temperature profiles (A, B, and C). In this plot, the crustal thickness is assumed to be 25 km, with the exception of temperature profile B that also considers a 50 km crust.

We note that our simulations with a melt layer are not entirely self-consistent with the temperature profiles in Supplementary Fig. 1. Our approach was to investigate the consequence of adding a melt layer at the base of the crust while keeping all other variables constant. Given that the composition of the melt layer is uncertain, estimating its liquidus and solidus temperatures would also be uncertain. Furthermore, the composition of the melt layer changes as the magma ocean continues to crystallize. To investigate how a melt layer would affect the basin morphology, we thus simply changed the viscosity of the material to a low, non-zero, value (100 Pa s), that is appropriate for molten materials within magma oceans[11] while leaving the temperature of the melt unchanged.

The list of input parameters for our iSALE simulations is shown in Supplementary Table 2. These models are very similar to the models used extensively in previous lunar basin modelling[8,10,12–18]. Our previous work[10] investigated the effects of acoustic fluidisation on large crater formation and found that its inclusion had little to no influence on the final basin morphology. In that study, the basin depth was largely unchanged when using acoustic fluidization with warm and hot temperature profiles in the target. However, it was shown that it was necessary to include acoustic fluidisation in order to match the observed depth of the basin floor when using colder temperature profiles. Acoustic fluidisation is included in this work because temperature profile B is comparable to some of the cold profiles used in Miljkovic et al.[10]. Acoustic fluidisation was applied in the same way on targets both with and without a melt layer. Furthermore, for the simulations that included a melt layer, test simulations were made with acoustic fluidisation turned on and off, which demonstrated that the final crater morphology was insignificantly different for the two cases. This further establishes that the melt layer is the dominant factor that controls the final crater morphology in our simulations (Supplementary Fig. 3c).

In our simulations, we used both 17 km/s and 10 km/s for the bolide impact speed. The two speeds did not show significant differences in basin morphologies when the outcomes are analyzed in terms of the kinetic energies of the impactor. We chose to focus on the 17 km/s impact speed in order to limit the number of free parameters in our simulations. Simulations were run until the crater modification stage was completed, which was confirmed by the crater profile (both at the surface and the interface between the crust and the mantle) reaching a stable position and not moving more than a couple of cells over a significant time interval. This equilibrium was reached typically within ~3 h following impact depending on the basin size, which is similar to previous works[10,13–17,25].

In our work, we compare the relief of the surface and crust-mantle interface with the known surface topography of the Moon and GRAIL-derived crustal thickness models (Model 1 of ref. 2). An alternative approach could have been instead to compare directly the gravity field predicted by iSALE with GRAIL observations. However, to do so, it would have been necessary to specify the density and porosity of the crust, which are both uncertain. Any undamped numerical oscillations in the vertical direction at the end of the iSALE simulations would also have affected the gravity signal. We note that isostatic adjustment of the basin (which is largely vertical in nature) would modify the predicted gravity field but would not affect significantly the crustal thickness. For these reasons, we chose to the analyse the crustal thickness profiles instead of the predicted gravity, as they are less affected by the details of how the gravity was computed.

In this work, we focus on the final crustal thickness variations, and the formation of basin rings and their spacing. Though the final surface relief is also an important outcome of basin formation, this was not easy to interpret for many of our iSALE simulations. For example, for the same sized impact basin, the cell dimension was 2.5 km by 2.5 km when basin formation was modelled until completion, but we used 500 by 500 m cells when simulating the formation of faults (rings). Many of our highest resolution simulations that focused on faulting did not run to completion because of computational resource considerations. In simulations where the melt layer was present, even after 3 h following basin formation, there were vertical oscillations (equal to a couple of cells moving up-down) that affected equally the surface and crust-mantle interface, but not the crustal thickness that is the difference of the two. For this reason, we did not interpret the final surface relief predicted by our models, but concentrated instead almost exclusively on the resulting crustal

thickness profile. We note that in this study, we made between 150 and 200 simulations all of which required day to weeks-long runtimes.

Supplementary Table 2. Summary of chosen numerical input parameters for the target layers: ROCK is the strength model[19], IVANOV is the damage model[20], BLOCK is the acoustic fluidisation model[21], and OHNAKA is the rock softening model[22]. The analytical equations of state were those of granite[23] for the crust and dunite for the mantle and melt layer. The material parameters used for the mantle are from Ivanov et al[24].

Description	Crust	Melt layer	Mantle
Equation of state	Granite ANEOS	Dunite ANEOS	Dunite ANEOS
Rock strength model	ROCK	Liquid	ROCK
Damage model	IVANOV	IVANOV	IVANOV
Acoustic fluidisation model	BLOCK	BLOCK	BLOCK
Thermal softening model	OHNAKA	OHNAKA	OHNAKA
Poisson ratio	0.3	0.5	0.25
Strength at zero pressure (intact)	10 MPa	0	50 MPa
Limiting strength	2500 MPa	0	3500 MPa
Internal friction coefficient (intact)	1.1	0	1.5
Strength at zero pressure (damaged)	0.01 MPa	0	0.01 MPa
Internal friction coefficient (damaged)	0.8	0	0.6
Melt temperature	1673 K	0	1436 K
Specific heat	1196 J/kg/K	1217 J/kg/K	1217 J/kg/K
Thermal softening parameter	1.2	0	2
Simon parameter A	6000	0	1400
Simon parameter B	3	0	5
Viscosity	-	100 Pa s	-
Ivanov parameter A	10^{-4}	10^{-4}	10^{-4}
Ivanov parameter B	10^{-11}	10^{-11}	10^{-11}
Ivanov parameter C	3×10^{8}	3×10^{8}	3×10^{8}
Viscosity of the acoustic fluidisation	1.8×10^{6}	0	1.8×10^{6}
Decay time for block oscillations	2070 s	0	2070 s
Acoustic fluidisation: viscosity scaling parameter	0.008	0	0.008
Acoustic fluidisation: decay time scaling parameter	230	0	230

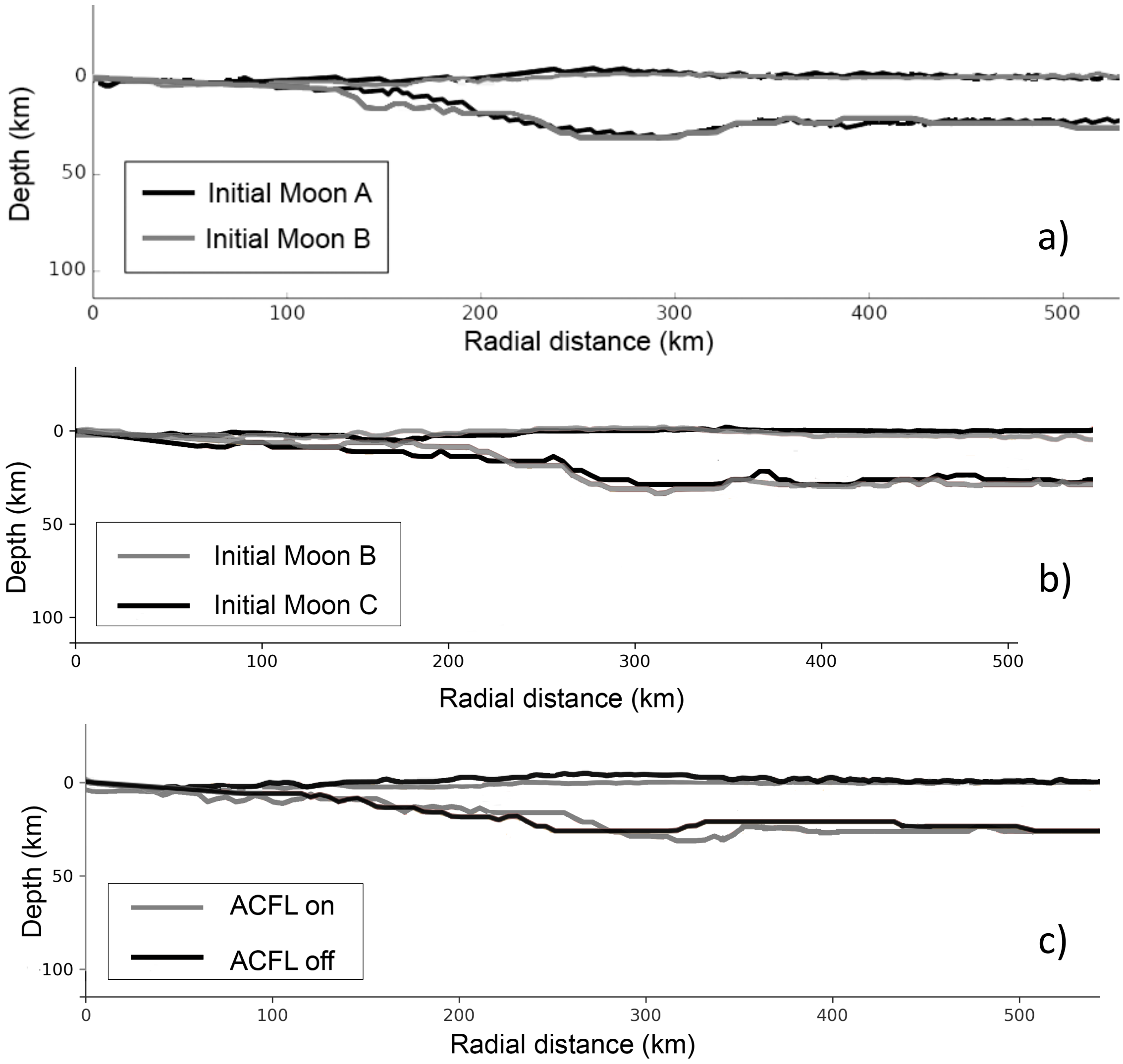

Supplementary Fig. 3: Parameter sensitivity tests showing that the temperature profile and acoustic fluidization do not affect significantly the final basin morphology when a melt layer is present. The simulations were made using a 60 km impactor onto a 25 km thick crust that overlays a 25 km thick melt layer and solid mantle. In each panel, the elevations of the surface and crust-mantle profile are plotted for two contrasting simulations. (a) Final basin morphology using temperature profiles A and B. (b) Final basin morphology using temperature profiles B and C. (c) Final basin morphology using temperature profile B both with and without acoustic fluidization. In all cases, there is little difference in the final basin morphology for the tested parameters.

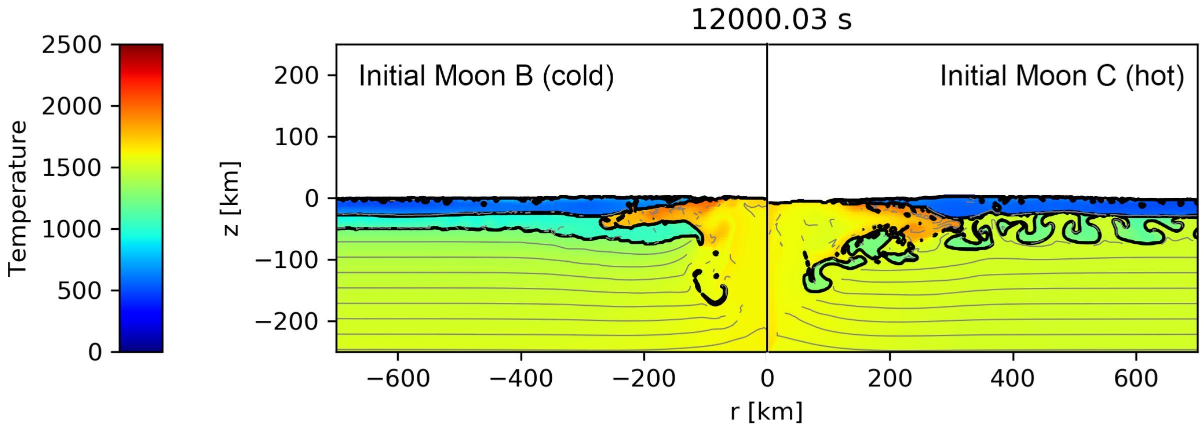

Supplementary Fig. 4: Temperature (in Kelvin) at the end of two iSALE simulations that included a 25 km thick melt layer using two initial temperature profiles, B (left) and C (right). The impactor diameter was 60 km impactor, the impact speed was 17 km/s, and the crustal thickness was 25 km. Heavy black contours delimit the crust, initial melt layer and mantle boundaries, whereas the thin connected lines denote initially horizontal tracer particles that were separated by a 20-km depth interval. The impact melt region is approximated by temperatures greater than 1500 K. Tracers were removed from the simulations when they become too distorted, and the central region that lacks tracer lines closely corresponds to the region of impact melted materials. The impact melted region is roughly bowl-shaped, is no more than 200 km deep in the basin centre, and extends up to 300 km radially at the surface level. Most of the impact melted materials are derived from the mantle. Though the impact melt pool is slightly larger in the hot case, the total impact melt volume and geometry are similar for the two temperature profiles.

Supplementary Note 2: Numerical impact modelling: Results

Supplementary Note 2.1: Structural features (ring formation) in basins

Supplementary Fig. 5 shows a moment during the modification phase of basin formation when large-scale faulting takes place and rings form. This figure corresponds to the formation of a Nectaris/Orientale-sized basin into a 25 km thick crust where the thickness of the melt layer is (a) 0 km (b) 10 km, (c) 25 km, and (d) 50 km. For this basin size, when there is no melt present, faults propagate and are focused in the regions of the peak ring (~250 km radial distance) and main rim/ring (~420 km radial distance). These locations agree with the observed rings of the Orientale and Nectaris basins (Supplementary Table 1 and Johnson et al.[8]). As the thickness of the melt layer increases, faults that initiate at depth and propagate to the surface become progressively hindered as a result of the melt layer that decouples stresses in the mantle and crust.

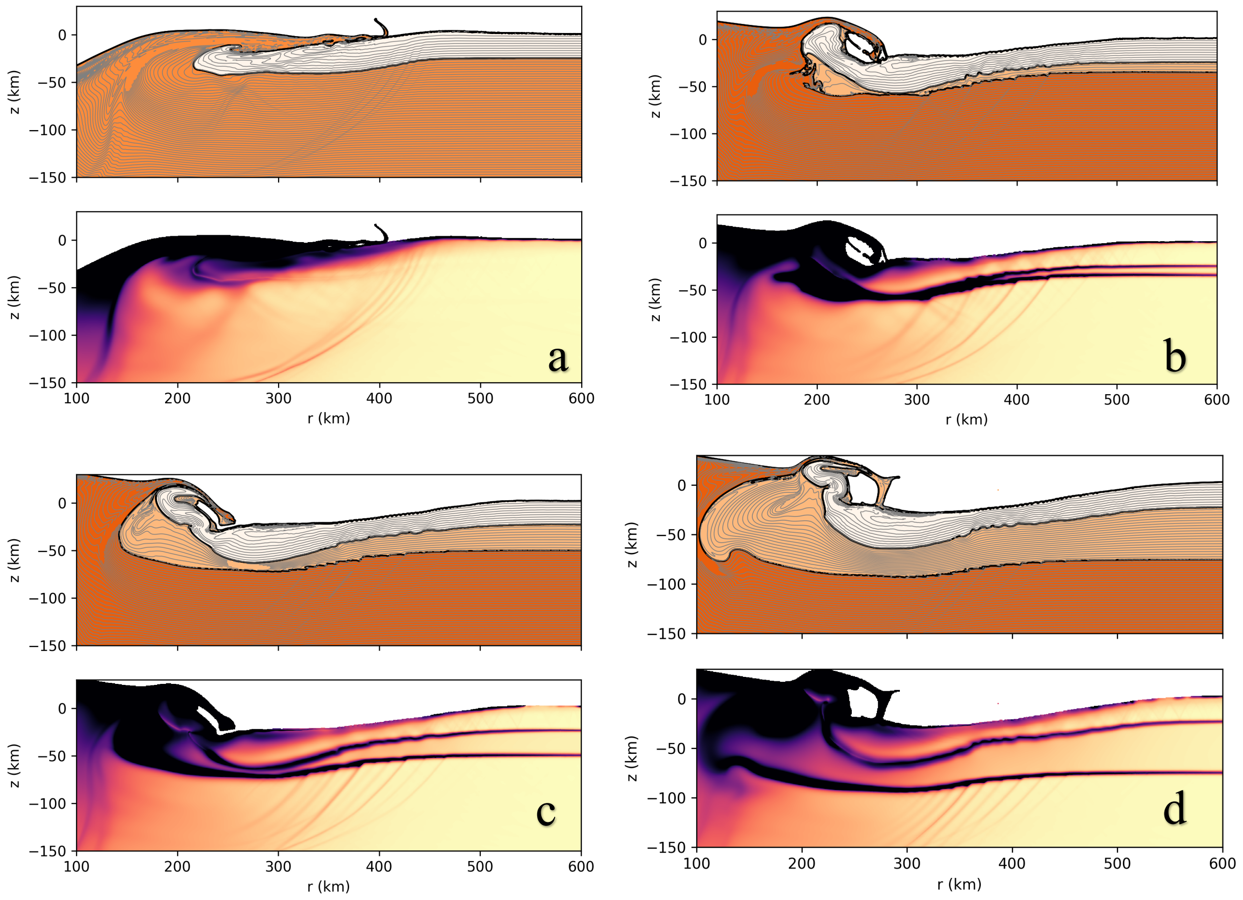

Supplementary Fig. 5: Ring formation during the crater modification stage for the cases where the melt layer thickness is (a) 0 km, (b) 10 km, (c) 25 km, and (d) 50 km, all with a 25-km thick crust. The impactor diameter was 60 km and the impact speed was 17 km/s. This figure includes the results presented in Fig. 3 for a melt layer thickness of 25 km.

Supplementary Note 2.2: Basin morphology with respect to basin size

Supplementary Fig. 6 shows the impact basin morphology for a wide range of basin sizes (from as small as ~200 km in diameter up to the 2000-km diameter South Pole-Aitken sized basin). Panels (a) to (e) are arranged with increasing basin size, and together they demonstrate that as long as there is a melt layer present (panels on the right), the final basin morphology is significantly different than when no melt layer present (panels on the left). For these examples, the melt layer thickness is set to 25 km. When a melt layer is present, the smaller basins tend not to form the crustal thickening surrounding the crustal thinning, but instead show a more gradual crustal thickness profile. For basins that are as large as the South Pole-Aitken basin, the effects of including a melt layer are not as prominent when compared to the simulation results of smaller basins (namely in terms of crustal inflow and extent of the crustal thinning).

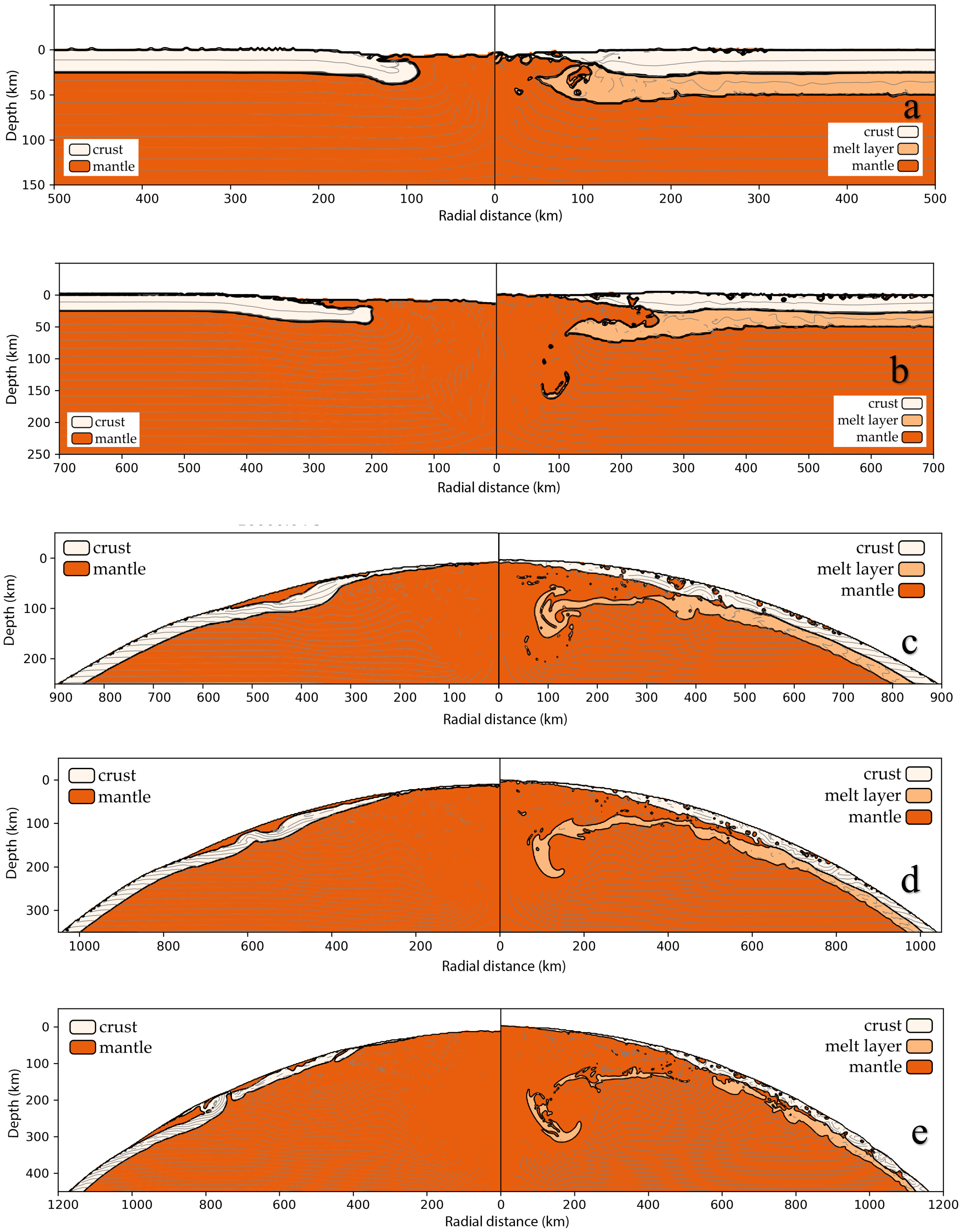

Supplementary Fig. 6: Change in basin morphology and stratigraphy with increasing basin size. Simulations correspond to projectile diameters of (a) 30, (b) 60, (c) 90, (d) 120 and (e) 160 km, the impact speed was 17 km/s, and the initial crustal thickness was 25 km. Images on the right include the presence of a 25 km thick melt layer, whereas the melt layer is absent on the left. Panels b and e were shown in Fig. 2. The final crater diameter (based on the location of the inner

rings and crustal thinning) for basins without the melt layer are approximately a) 300 km, b) 500 km, c) 700 km, d) 1000 km, and e) 1600 km. The uncertainty in these diameter estimates is about 10 km for the smallest crater, and up to 100 km for the largest. Diameters are not estimated for the case where a melt layer is present (right) given the lack of clearly identifiable crustal thickness characteristics and the multitude of faults in the crust.

Supplementary Note 2.3: Basin morphology with respect to the melt layer thickness

Supplementary Fig. 7 shows the final basin morphology as a function of melt layer thickness for Orientale and Nectaris sized basins, where the melt layer thickness is (a) 0 km, (b) 10 km, (c), 25 km, and (d) 50 km. The basin morphology is only weakly dependent on the thickness of the melt layer, when the melt layer is thicker than 10 km. For the case where there is no melt layer, a peak ring and main rim form at radial distances of ~220 and ~440 km, respectively. For the thinnest melt layer thickness of 10 km, it is possible that a single ring-like fault forms at the surface at a radial distance of ~300 km. However, any ring-like structure is ambiguous for thicker melt layers (panels C and D). This result is compatible with the observation that there is usually only one possible observable ring for the oldest pre-Nectarian basins (Supplementary Table 1).

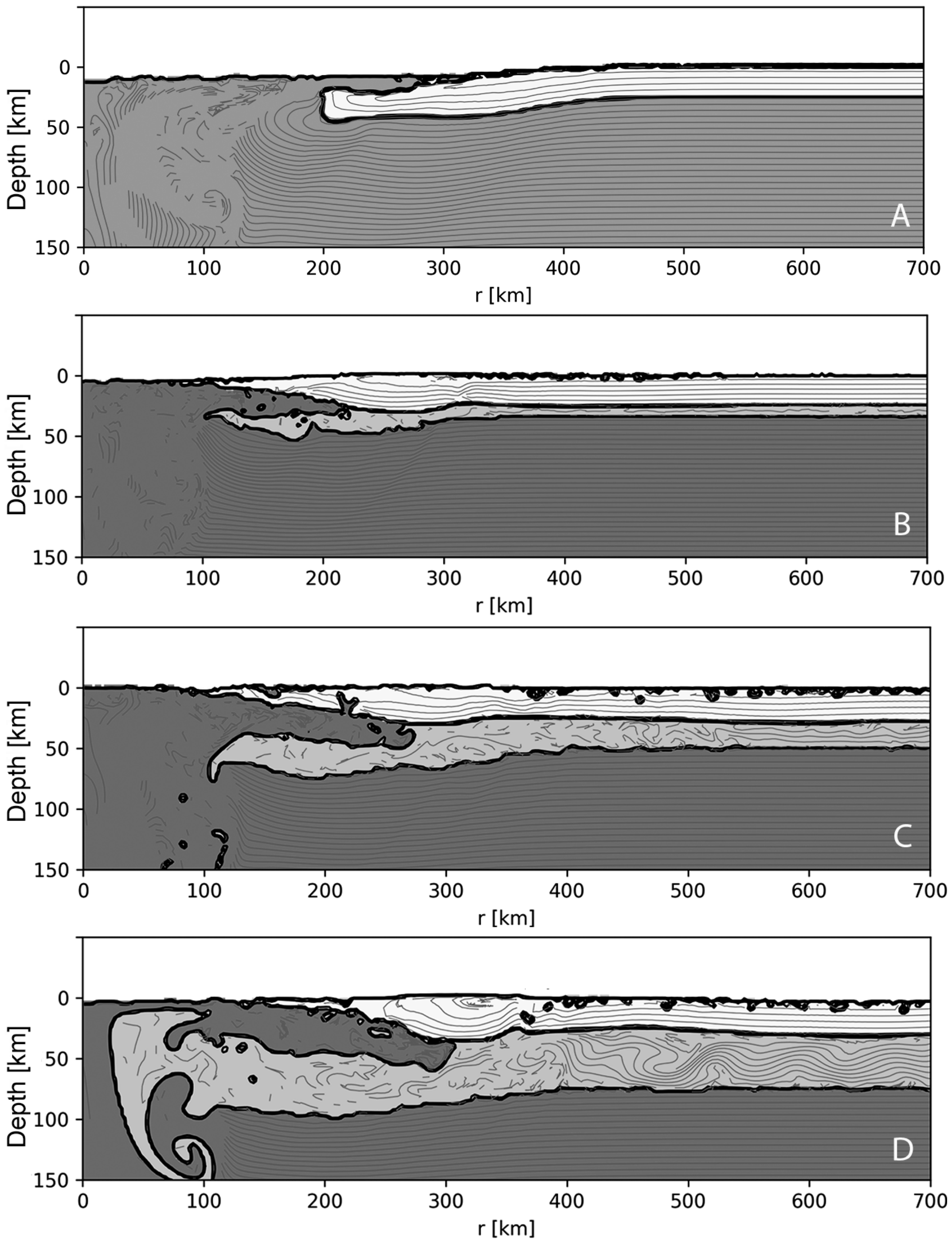

Supplementary Fig. 7: Basin morphology dependence on melt layer thickness. From top to bottom, the melt layer is 0, 10, 25, and 50 km thick. The projectile diameter was 60 km and impacted into a 25 km thick crust at 17 km/s. White, light grey and dark grey shades denote the crust, melt layer, and mantle, respectively. Massless tracer particles (denoted by connected lines) were placed initially at every 5 km depth in the pre-impact target.

Supplementary Note 2.4: Basin morphology with a melt layer and different crustal thicknesses

Supplementary Fig. 8 shows the morphology of an Orientale/Nectaris sized basin that formed onto a 10 km, 25 km, and 50 km thick crust, respectively. While the crustal morphology appears comparable between panels, a major difference is the amount of crustal inflow that occurs towards the basin centre. For the thickest initial crustal thickness (panel c), a continuous crustal cap is found to cover the entire basin interior. In contrast, for the thinnest initial crustal thicknesses, an exposed impact melt pool is present in the basin interior. For the intermediate case the crustal materials are discontinuous in the basin interior.

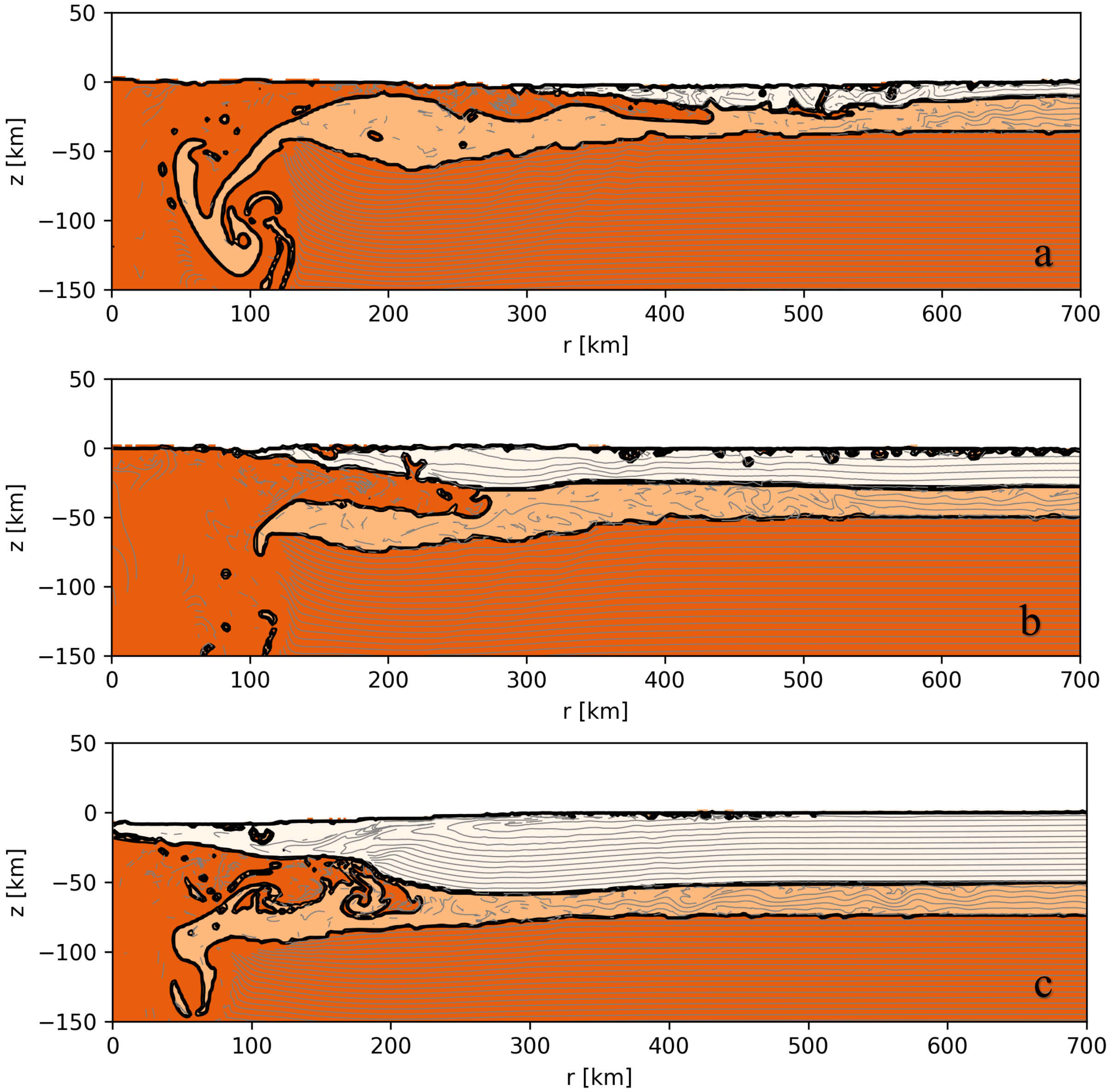

Supplementary Fig. 8: Basin morphology dependence on initial crustal thickness. From top to bottom, the initial crustal thickness was 10, 25, and 50 km. The melt layer was 25 km thick in all cases, with other conditions being the same as in Supplementary Fig. 5.

Supplementary Note 2.5: Basin crustal thickness signature as a function of basin size and melt layer thickness

Supplementary Fig. 9 shows crustal thickness signatures as a function of basin size and melt layer thickness. Each panel corresponds to a single projectile size (15, 30, 60, 90, 120 and 160 km diameter), and the crustal profiles correspond to different melt layer thicknesses, ranging from 0 (purple), 10 km (blue), 25 km (grey), to 50 km (red). When no melt layer is present, all basins show a prominent thinning of the crust in the basin interior. When the melt layer is >10 km, all basins show a very similar profile, where the crustal thickness is larger in the basin interior and grades more smoothly to the ambient pre-impact value than for the case where no melt layer is present.

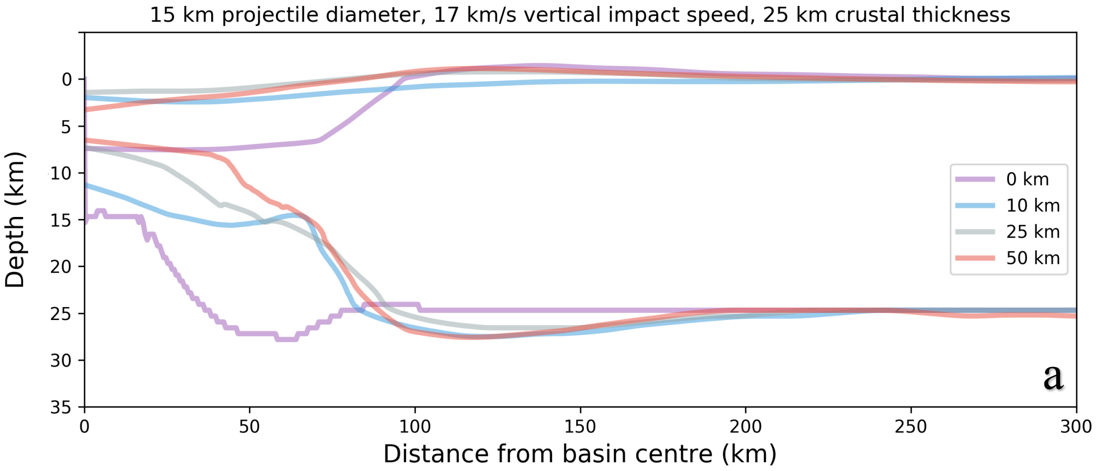

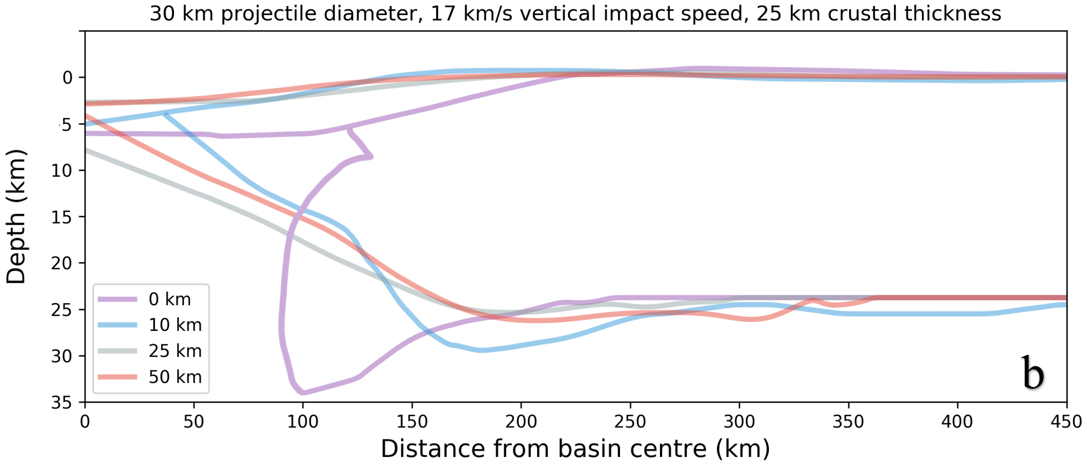

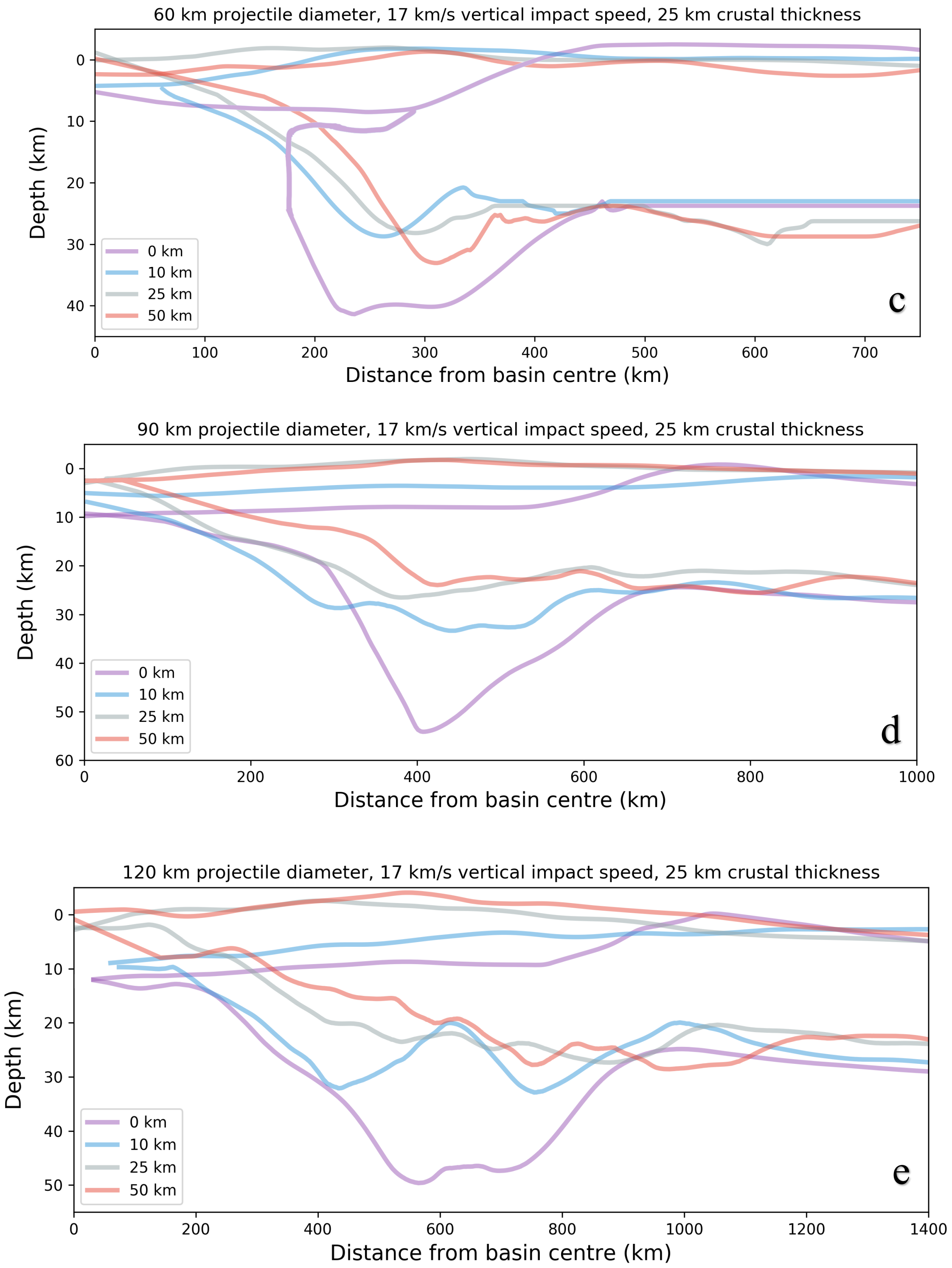
60 km projectile diameter, 17 km/s vertical impact speed, 25 km crustal thickness
Depth (km)
0
10
20
30
40
0
100
200
300
400
500
600
700
Distance from basin centre (km)
0 km
10 km
25 km
50 km
c
90 km projectile diameter, 17 km/s vertical impact speed, 25 km crustal thickness
Depth (km)
0
10
20
30
40
50
60
0
200
400
600
800
1000
Distance from basin centre (km)
0 km
10 km
25 km
50 km
d
120 km projectile diameter, 17 km/s vertical impact speed, 25 km crustal thickness
Depth (km)
0
10
20
30
40
50
0
200
400
600
800
1000
1200
1400
Distance from basin centre (km)
0 km
10 km
25 km
50 km
e

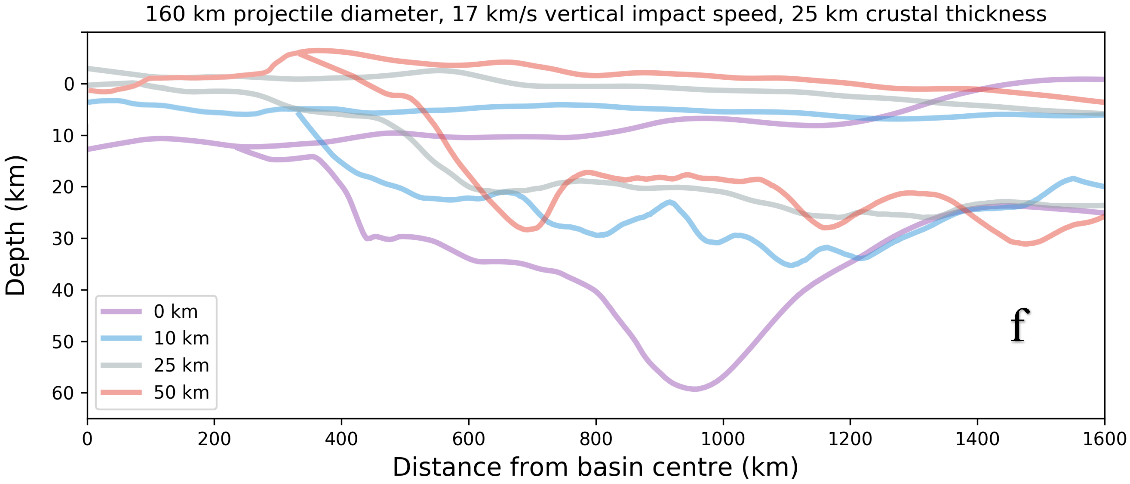

Supplementary Fig. 9: Crustal profiles for the entire range of basin sizes on the Moon for melt layer thicknesses of 0 km (purple), 10 km (blue), 25 km (grey) and 50 km (red). Each panel corresponds to a single projectile diameter impacting a 25 km thick crust at 17 km/s. From top to bottom, the projectile diameter increases from 15 km to 160 km. Panel (c) corresponds to an Orientale-sized basin, and panel (f) to a South Pole-Aitken-sized basin. Top lines represent the surface, and bottom lines represent boundaries between the crust and underlying material. The confidence level for these crustal profiles is 2 numerical cells, which corresponds to 1.3 km for the smallest basins (a) to 5 km for the largest (e-f).

Supplementary Note 2.6: Best-fit scenario for the formation of the South-Pole Aitken basin

The South Pole-Aitken basin is thought to be the largest and oldest impact basin on the Moon[7]. Our best-fit simulation for this basin was for a 160 km diameter projectile, similar to what was used in previous studies[7]. When a melt layer is present (of any thickness larger than 10 km), we find extensive crustal inflow into the basin centre giving rise to a crustal cap covering the entire impact melt sheet. When no melt layer is present, similar to previous investigations, basin formation ends with an exposed mantle-rich melt pool at the surface[7]. Although, the large size of the South Pole-Aitken basin in comparison to the lunar lithospheric thickness may explain the lack of prominent inner and outer ring structures, the final crustal signature when including a melt layer is the most similar to the GRAIL-derived crustal thickness profile (Supplementary Fig. 10)[2]. Our simulations suggest that the crustal inflow is composed mostly of broken rafts flowing back into the basin centre that are composed primarily of overturned and jumbled crust originating from lower and mid-level crust levels.

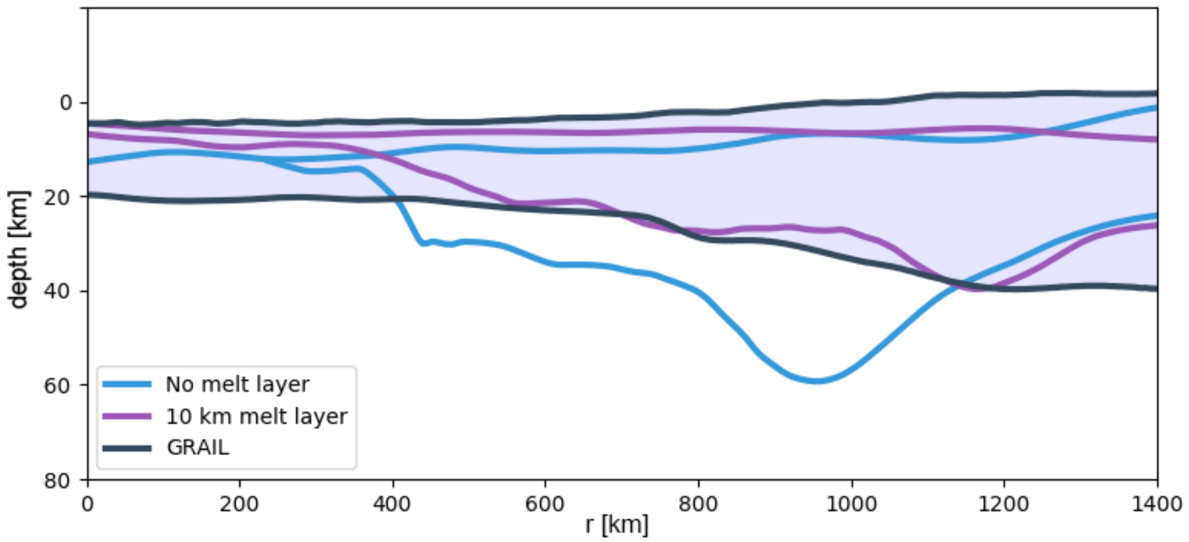

Supplementary Fig. 10: GRAIL-derived azimuthally averaged crustal thickness profile for the South Pole-Aitken (SPA) basin (black, with crustal thickness shaded in pale purple) compared to profiles obtained from two iSALE simulations (purple and blue). The simulations used a 160-km diameter projectile striking the Moon at 17 km/s, with and without a 10-km thick melt layer. The pre-set choice of crustal thickness, here shown at 25 km, produced slightly thinner crustal profile than what was observed at SPA.

Supplementary References:

1. Neumann, G. A. *et al.* Planetary Science: Lunar impact basins revealed by gravity recovery and interior laboratory measurements. *Sci. Adv.* **1**, (2015).

2. Wieczorek, M. A. *et al.* The crust of the moon as seen by GRAIL. *Science (80-.).* **339**, 671–675 (2013).

3. Elkins-Tanton, L. T. & Bercovici, D. Contraction or expansion of the Moon's crust during magma ocean freezing? *Philos. Trans. R. Soc. A Math. Phys. Eng. Sci.* **372**, (2014).

4. Elkins-Tanton, L. T., Burgess, S. & Yin, Q. Z. The lunar magma ocean: Reconciling the solidification process with lunar petrology and geochronology. *Earth Planet. Sci. Lett.* **304**, 326–336 (2011).

5. Solomatov, V. S. & Stevenson, D. J. Nonfractional crystallization of a terrestrial magma ocean. *J. Geophys. Res.* **98**, 5391–5406 (1993).

6. Reese, C. C. & Solomatov, V. S. Fluid dynamics of local martian magma oceans. *Icarus* **184**, 102–120 (2006).

7. Potter, R. W. K., Collins, G. S., Kiefer, W. S., McGovern, P. J. & Kring, D. A. Constraining the size of the South Pole-Aitken basin impact. *Icarus* **220**, 730–743 (2012).

8. Johnson, B. C. *et al.* Formation of the Orientale lunar multiring basin. *Science (80-.).* **354**, (2016).

9. Laneuville, M., Wieczorek, M. A., Breuer, D. & Tosi, N. Asymmetric thermal evolution of the Moon. *J. Geophys. Res. E Planets* **118**, 1435–1452 (2013).

10. Miljković, K. *et al.* Asymmetric distribution of lunar impact basins caused by variations in target properties. *Science (80-.).* **342**, 724–726 (2013).

11. Solomatov, V. S. Magma oceans and primordial mantle differentiation. in *Treatise on Geophysics* (ed. Schubert, G.) 91–120 (Elsevier, 2007).

12. Miljković, K. *et al.* Excavation of the lunar mantle by basin-forming events on the Moon. *Earth Planet. Sci. Lett.* **409**, 243–251 (2015).

13. Miljković, K. *et al.* Subsurface morphology and scaling of lunar impact basins. *J. Geophys. Res. Planets* **121**, 1695–1712 (2016).

14. Miljković, K., Lemelin, M. & Lucey, P. G. P. G. Depth of Origin of the Peak (Inner) Ring in Lunar Impact Basins. *Geophys. Res. Lett.* **44**, 10,140-10,146 (2017).

15. Potter, R. W. K., Kring, D. A., Collins, G. S., Kiefer, W. S. & McGovern, P. J. Numerical modeling of the formation and structure of the Orientale impact basin. *J. Geophys. Res. E Planets* **118**, 963–979 (2013).

16. Potter, R. W. K., Kring, D. A., Collins, G. S., Kiefer, W. S. & McGovern, P. J. Estimating transient crater size using the crustal annular bulge: Insights from numerical modeling of lunar basin-scale impacts. *Geophys. Res. Lett.* **39**, 1–5 (2012).

17. Potter, R. W. K., Kring, D. A. & Collins, G. S. Scaling of basin-sized impacts and the influence of target temperature. **2518**, 99–113 (2015).

18. Johnson, B. C. *et al.* Controls on the formation of lunar multiring basins. *J. Geophys. Res. Planets* **123**, 3035–3050 (2018).

19. Collins, G. S., Melosh, H. J. & Ivanov, B. A. Modeling damage and deformation in impact simulations. *Meteorit. Planet. Sci.* **39**, 217–231 (2004).

20. Ivanov, B. A., Deniem, D. & Neukum, G. Implementation of dynamic strength models into 2D hydrocodes: Applications for atmospheric breakup and impact cratering. *Int. J. Impact Eng.* **20**, 411–430 (1997).

21. Melosh, H. J. & Ivanov, B. A. Impact Crater Collapse. *Annu. Rev. Earth Planet. Sci.* **27**, 385–415 (1999).

22. Ohnaka, M. A shear failure strength law of rock in the brittle-plastic transition regime. *Geophys. Res. Lett.* **22**, 25–28 (1995).

23. Pierazzo, E., Vickery, A. M. & Melosh, H. J. A reevaluation of impact melt production. *Icarus* **127**, 408–423 (1997).

24. Ivanov, B. A., Melosh, H. J. & Pierazzo, E. Basin-forming impacts: Reconnaissance modeling. *Spec. Pap. Geol. Soc. Am.* **465**, 29–49 (2010).

25. Miljković, K. *et al.* Excavation of the lunar mantle by basin-forming events on the Moon. *Earth Planet. Sci. Lett.* **409**, (2015).
